# Tissue factor expression as a possible determinant of thromboembolism in ovarian cancer

**DOI:** 10.1038/sj.bjc.6603552

**Published:** 2007-01-09

**Authors:** K Uno, S Homma, T Satoh, K Nakanishi, D Abe, K Matsumoto, A Oki, H Tsunoda, I Yamaguchi, T Nagasawa, H Yoshikawa, K Aonuma

**Affiliations:** 1 Cardiovascular Division, Institute of Clinical Medicine, Graduate School of Comprehensive Human Science, University of Tsukuba, 1-1-1 Tennodai, Tsukuba, Ibaraki, Japan; 2 Department of Obstetrics and Gynecology, Institute of Clinical Medicine, Graduate School of Comprehensive Human Science, University of Tsukuba, Tsukuba, Ibaraki, Japan; 3 Department of Clinical and Experimental Hematology, Major of Advanced Biomedical Applications, Graduate School of Comprehensive Human Science, University of Tsukuba, Tsukuba, Ibaraki, Japan

**Keywords:** ovarian cancer, clear cell carcinoma, venous thromboembolism, tissue factor

## Abstract

Ovarian cancer, and clear cell carcinoma in particular, reportedly increases the risk of venous thromboembolism (VTE). However, the mechanisms remain unclear. Tissue factor (TF) supposedly represents a major factor in the procoagulant activities of cancer cells. The present study examined the involvement of TF expression in VTE for patients with ovarian cancer. Subjects comprised 32 consecutive patients (mean age 49.8 years) with histologically confirmed ovarian cancer. Presence of VTE was examined using a combination of clinical features, D-dimer levels and venous ultrasonography. Immunohistochemical analysis was used to evaluate TF expression into 4 degrees. Venous thromboembolism was identified in 10 of the 32 patients (31%), including five of the 11 patients with clear cell carcinoma. Tissue factor expression was detected in cancer tissues from 24 patients and displayed significant correlations with VTE development (*P*=0.0003), D-dimer concentration (*P*=0.003) and clear cell carcinoma (*P*<0.05). Multivariate analysis identified TF expression as an independent predictive factor of VTE development (*P*<0.05). Tissue factor (TF) expression is a possible determinant of VTE development in ovarian cancer. In particular, clear cell carcinoma may produce excessive levels of TF and is more likely to develop VTE.

Venous thromboembolism (VTE) is a common complication of cancer (Baron *et al*, 1998; Lee and Levine, 2003). Since the first description of an association between VTE and cancer by Trousseau (1868), cancer has emerged as an important risk factor for VTE. A prospective medical database of a county population in the United States reported cancer alone as associated with a 4.1-fold increase in risk of VTE, compared to the annual incidence of a first episode of VTE in the general population of 117 out of 100 000-capita (Silverstein *et al*, 1998; Heit *et al*, 2000). Conversely, Sørensen *et al* (2000) reported that 1-year survival rate was significantly lower in cancer patients with VTE (12%) than in cancer patients without VTE (36%), and the mortality ratio associated with VTE was 2.2 for the 1-year follow-up period. This mortality probably reflects deaths owing to VTE, and VTE complication may thus cause a life-threatening condition in cancer patients. To improve survival rate for cancer patients, clarification of the clinical characteristics and pathogenic mechanisms of VTE with cancer is necessary.

Ovarian cancer is known to display a particular association with VTE (Heit *et al*, 2000; von Tempelhoff *et al*, 2000). According to data from Medicare International Classification of Disease, 9th Revision (ICD-9) hospital discharge diagnoses between 1988 and 1990 in the United States, ovarian cancer exhibited the highest incidence of cancer-related VTE, at 120 out of 10 000 patients (Levitan *et al*, 1999). Moreover, some reports have suggested that clear cell carcinoma of the ovary is particularly likely to develop VTE (Pather and Quinn, 2005). Yoonessi *et al* (1984) reported that 13.7% of patients with clear cell carcinoma of the ovary developed thromboembolic complications. Recio *et al* (1996) reported an 11% incidence of symptomatic thromboembolic complications with clear cell ovarian carcinoma. Although ovarian cancer, and clear cell carcinoma in particular, seems more likely to result in VTE, the pathological mechanisms remain speculative.

Regarding the mechanisms underlying VTE formation in cancer patients, several tumour cell procoagulant activities have been identified that may act at steps in blood coagulation pathways (De Cicco, 2004). Numerous studies have suggested that tissue factor (TF) may play an important role in the pathogenesis of hypercoagulable states in patients with cancer (Rao, 1992), and TF expression has been confirmed in some cancer tissues by immunohistochemistry (Callander *et al*, 1992; Ueno *et al*, 2000). However, correlations between TF expression in cancer tissues and clinical VTE formation have not yet been proven.

The purpose of this study was to clarify the involvement of TF expression in VTE with ovarian cancer, particularly clear cell carcinoma. We also investigated relationships between TF expression and D-dimer concentration, as a marker of procoagulant activity.

## MATERIALS AND METHODS

### Study population

All study protocols were approved by the Ethical Committee of Tsukuba University Hospital. The study complied with the principles of the Declaration of Helsinki. A total of 36 consecutive patients with ovarian cancer admitted for initial treatment to the Department of Gynecology and Obstetrics, Tsukuba University Hospital, were enrolled in this study from January 2004. Only patients with newly diagnosed ovarian cancer were included. All patients provided written informed consent to participate in the study. Patients were excluded if clinically significant renal, hepatic or cardiac disease was present. A medical history was taken for thromboembolic risk factors including: age, body mass index (BMI), hypertension, diabetes mellitus, venous varicosities, preoperative immobility and previous history of VTE. Medication use was also documented for each patient, including oral hormonal drugs. Clinical stages were determined according to the criteria of the International Federation of Gynecology and Obstetrics (FIGO), and histological classifications were also evaluated. Haematological analysis and VTE diagnosis were performed before any treatment. All patients underwent gynaecological surgery, and immunohistochemical analysis was performed with surgical tissue with the exception of biopsy tissue before neoadjuvant chemotherapy. And all assessments including ultrasonography, immunohistochemical and pathological diagnoses, were evaluated blinded to their diagnoses and haematological analysis.

### Haematological analysis

Before treatment, peripheral blood samples were collected from all patients and D-dimer levels were measured. Blood samples were drawn from an antecubital vein with atraumatic puncture into plastic tubes using a two-tube technique, discarding the first 4–5 ml. Whole blood was anticoagulated with the addition of 9 volumes to 1 volume of 3.2% sodium citrate solution, then centrifuged at 3000 r.p.m. for 10 min. Citrate plasma was then removed and frozen at −20°C up to 3 days before assessment. D-dimer levels were measured by latex photometric immunoassay (Roche Diagnostics, Basel, Switzerland) (Kario *et al*, 1992).

### Diagnosis of VTE

Presence of VTE was identified according to procedures recommended elsewhere (Perrier *et al*, 1999; Andrews and Fleischer, 2000), using a combination of clinical features, D-dimer levels and venous ultrasonography. Ultrasongraphic examination of leg veins was performed for patients displaying symptoms of suspected VTE or highly elevated levels of D-dimer (⩾3.0 *μ*g ml^−1^). Ultrasonography was performed using an ATL HDI5000 system (PHILIPS Medical Systems, Bothell, WA, USA) equipped with a 3–7.5 MHz transducer according to the search site. The iliac, femoral, popliteal, peroneal, post-tibial and soleal veins were evaluated in both legs. Venous lumens were observed while searching for thrombus by manual compression with transducer and colour Doppler signals. For evaluation of intrapelvic veins, reactions during a Valsalva manoeuvre were also observed. No reaction during the Valsalva manoeuvre was considered to represent suspected proximal venous flow disturbance, and intrapelvic deep venous thrombosis (DVT) was diagnosed based on the results of enhanced computer tomography. In addition, lung perfusion scintigraphy was performed for patients with DVT, any symptoms suggesting pulmonary thromboembolism (PTE) or low oxygen saturation, to identify PTE.

### Immunohistochemistry

To examine the association between TF expression and VTE, we performed immunohistochemical assessment for all patients with or without VTE. Surgical specimens were fixed in 10% formalin and embedded in paraffin. Each tumour specimen was stained on ⩾3 separate occasions. Thin sections (3 *μ*m) were prepared, deparaffinised with xylene and rehydrated in ethanol, then endogenous peroxidase was blocked in 3% H_2_O_2_ in methanol for 15 min. After washing in water, all sections were irradiated by microwave for 5 min in 10 mM citrate buffer (pH 6.0). For the immunoperoxidase method, sections were incubated with 1 : 50 diluted anti-human TF antibody (4509; American Diagnostica, Stamford, CT, USA) overnight at 4°C. Sections were further incubated in biotinylated horse anti-mouse immunoglobulin-G (IgG) (Vector ABC elite kit, Vector Laboratories, Burlingame, CA, USA), followed by avidin–biotin–peroxidase complex (ABC; Vector, Burlingame, CA, USA). Both biotinylated IgG and ABC solutions were prepared with phosphate-buffered saline containing 0.5% skim milk to eliminate the background staining. Immunoreactions were visualised using diaminobenzidine tetrahydrochloride.

For each set of staining experiments, positive and negative controls were used. The positive control comprised a section of umbilical cord, which is known to stain brightly for TF (Callander *et al*, 1992), overlaid with anti-human TF IgG. Sections incubated with normal mouse serum instead of the primary antibody served as negative controls.

**Figure 1:**
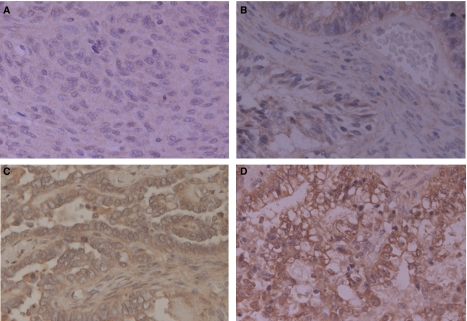
Immunohistochemical staining of tissue factor in ovarian carcinoma tissues. (**A**) Negative control, (−); (**B**) weakly positive (<50% positive tumour cells), (+); (**C**) moderately positive (⩾50% positive tumour cells with weak intensity), (++); (**D**) strongly positive (⩾50% positive tumour cells with strong intensity), (+++), based on the proportion of the entire tumour cell population positive for TF. All pictures were taken at original magnification × 200.

Intensity of TF expression was classified into four degrees as follows: (−), negative; (+), weakly positive (<50% positive tumour cells); (++), moderately positive (⩾50% positive tumour cells with weak intensity); (+++), strongly positive (⩾50% positive tumour cells with strong intensity), based on the proportion of the entire tumour cell population showing positive for TF (Hamada *et al*, 1996). Representative examples of each score are presented in Figure 1. To diagnose with the tissue after neoadjuvant chemotherapy, we used the region containing enough amount of their original cancer cell structure to be evaluated for pathological and immunohistochemical diagnoses. All immunohistochemical and pathological assessments were performed by three independent observers blinded to clinical conditions.

### Statistical analysis

To identify any prevalence effect for VTE development, Mann–Whitney's *U*-test was used for age and BMI, Spearman's correlation coefficient by rank was used for stage and TF expression, and Fisher's exact test (two-tailed) was used for histological classification (clear cell carcinoma or not). For multivariate analysis, logistic regression analysis was used to examine the effects of TF expression on VTE development after controlling for other clinically important risk factors such as age, BMI, stage and histological classification.

To examine correlations with TF expression, Spearman's correlation coefficient by rank was used for age, BMI, D-dimer level and stage, and Mann–Whitney's *U*-test was used for histological classification and presurgical chemotherapy.

Results are expressed as mean values (±s.d.) and values of *P*<0.05 were considered statistically significant.

## RESULTS

### Subject characteristics

A total of 36 consecutive untreated patients with stage I–IV ovarian cancer were managed in this study period. Of these, ovarian cancer could not be histologically confirmed in four patients owing to damage from neoadjuvant chemotherapy, and the remaining 32 patients with histological confirmed ovarian cancer were enrolled in the final analysis. None of these 32 patients had a previous history of VTE or use of hormonal drugs. Four patients had received pharmacotherapy for hypertension and two patients had received medical treatment for diabetes mellitus. Table 1 shows patient characteristics. Mean age on initial treatment was 49.8±13.7 years (range 18–77 years) and mean BMI was 23.3 ± 4.2 (range 18.3–39.4). According to the findings at major surgery, seven patients had stage I ovarian cancer, six patients stage II, 11 patients stage III and eight patients stage IV. Ovarian cancer specimens included 12 serous carcinomas, four endometrioid carcinomas, 11 clear cell carcinomas, three undifferentiated carcinomas, one mixed epithelial carcinoma and one yolk sac tumour.

Before treatment, 14 patients underwent ultrasonographic evaluation for VTE with abnormal D-dimer levels (9.59 ± 7.27 *μ*g ml^−1^; Table 1), and three of these patients showed symptoms of DVT, with leg pain in two patients and leg oedema in one patient. None of the patients without abnormal D-dimer elevation showed any symptoms. Of the 14 patients who underwent ultrasonography, 10 patients (31%) developed DVT in the leg veins. Pulmonary thromboembolism (PTE) was identified in four of the 10 patients (one symptomatic; three asymptomatic). No patients showed any symptom of suspected VTE in the absence of elevated D-dimer levels. Deep venous thrombosis was located proximal to the popliteal vein in four patients, but was limited to the calf veins in the remaining six patients. None of the 10 patients died from VTE, although one patient died of cancer during the observational period.

### Immunohistochemical study

Seventeen patients received neoadjuvant chemotherapy. Immunohistochemical analysis was performed with biopsy tissue before neoadjuvant chemotherapy for two patients, and surgical tissue after neoadjuvant chemotherapy for 15 patients.

A heterogeneous and studded pattern of TF staining was observed within sections (Figure 1). Specimens were thus evaluated semiquantitatively by calculating the percentage of positively stained neoplastic cells, as described in Materials and Methods. Tissue factor expression was detected in cancer tissues of 27 patients, including nine (+), 10 (++) and eight (+++).

Tissue factor expression, demographic characteristics, results of haematological examination, chemotherapy before surgery, stage, and histological classification of subjects are summarised in Table 2. A significant positive correlation between D-dimer level and TF expression was observed (*P*=0.0030, *ρ*=0.533). No significant correlations were noted between TF expression and age, BMI or stage. Tissues from clear cell carcinoma exhibited significantly stronger TF expression than tissues from non-clear cell carcinoma (*P*<0.05).

Table 3 shows the comparison between patients with and without VTE. A significant correlation was noted between intensity of TF expression and occurrence of VTE (*P*=0.0003, *ρ*=0.653). No significant differences were detected in age, BMI or stage. Frequency of VTE was significantly higher in patients with clear cell carcinoma (five of 11 patients; 45%) than in with non-clear cell carcinoma patients (five of 21 patients; 24%). However, no significant prevalence effect on VTE was identified for clear cell carcinoma (*P*=0.2515). Multivariate analysis detected age and TF expression as significant determinant risk factors for VTE development (*P*=0.0352, *r*=0.247 and *P*=0.0347, *r*=0.249, respectively).

## DISCUSSION

In this study, at least 31% (10 out of 32) of ovarian cancer patients displayed DVT (symptomatic, *n*=3; asymptomatic, *n*=7) before treatment, and 13% (four out of 32) had PTE (symptomatic, *n*=1; asymptomatic, *n*=3). Tissue factor expression was significantly stronger in patients with VTE than in patients without VTE (*P*=0.0003). Moreover, age and TF expression were also detected as significant determinant risk factors for VTE (*P*=0.0352 and *P*=0.0347, respectively) in multivariate analysis. Tissue factor expression was significantly increased in the presence of elevated D-dimer concentration (*P*=0.003). In addition, clear cell carcinoma (*n*=11) showed a high frequency of VTE (five out of 11; 45%) and significantly stronger TF expression than non-clear cell carcinoma (*P*<0.05). These results suggest that development of VTE may be mediated by TF expression, and TF expression was marked in clear cell carcinoma.

Compared to previous studies, pretreatment frequency of DVT in patients with ovarian cancer was high in the present investigation. In fact, the report by Sallah *et al* (2002) described symptomatic DVT in only four of 38 ovarian cancer patients (11%). Our study, however, focused on asymptomatic DVT patients and symptomatic DVT occurred in just three patients of the 32 patients, compared with asymptomatic DVT in seven of 32 patients. Symptomatic DVT patients have been known to be objectively far less common than in screening tests including asymptomatic DVT patients (Kassaï *et al*, 2004). Girard *et al* (1999) reported that 82% of patients with acute PTE still displayed detectable residual DVT at the time of PTE diagnosis, and DVT remains asymptomatic in nearly two-thirds of PTE patients. To prevent a clinical event such as PTE, identifying DVT in asymptomatic patients is important. To find DVT in asymptomatic patients, procedures using a combination of clinical features, D-dimer levels and venous ultrasonography have been recommended by various authors (Perrier *et al*, 1999; Andrews and Fleischer, 2000). D-dimer has been extensively investigated as a predictive value for DVT, and reportedly displays high sensitivity (83–100%) and negative predictive value (96–100%), although the specificity of D-dimer is known to be low (35–70%) (Perrier and Bounameaux, 2001; Righini *et al*, 2006). The sensitivity and specificity of ultrasonography for the diagnosis of DVT have been reported as 85–92 and 90–98%, respectively, and venous ultrasonography has been described as the most accurate noninvasive test for the diagnosis of DVT (Theodorou *et al*, 2003; Goodacre *et al*, 2005). A diagnostic algorithm using D-dimer level as an initial test to rule out DVT and ultrasonography in patients with abnormal D-dimer results has thus been proposed. This noninvasive combined method seems useful for detecting DVT in clinical situations.

Tissue factor, a transmembrane receptor lipophilic phospholipoprotien with potent procoagulant activity, reportedly becomes constitutively expressed in some cancer tissues (Rao, 1992; De Cicco, 2004). Expression of TF on tumour cells, tumour-associated macrophages and endothelial cells is upregulated in response to cytokines, such as tumour necrosis factor and interleukin-1 (Grignani and Maiolo, 2000). Tissue factor initiates the extrinsic pathway of the coagulation cascade by binding and activating factor VII (FVII) and increasing the activity of activated FVII; these events allow catalysis of factor X (FX) to activated FX, in turn activating prothrombin to thrombin. For the mechanism of hypercoagulable state in cancer, Szczepanski *et al* (1988) reported that the procoagulant activities of cell extracts from gastric, colorectal and renal cancers were FVII-dependent and could be related to the presence of TF within cancer tissue. Although the procoagulant activity promoted by TF might be the important process for development of VTE in cancer patients, the correlation between TF expression and VTE incidence had not been provided in previous studies. The present investigation confirmed TF expression in ovarian cancer tissues and evaluated the intensity of TF expression semiquantitatively. We also searched for VTE including in asymptomatic patients using US, and confirmed for the first time that TF expression is significantly correlated with VTE development in clinical situations. Procoagulant activity with D-dimer elevation may be mediated by TF expression in ovarian cancer.

In addition, TF expression in cancer tissue has been suggested to enhance transcription of vascular endothelial growth factor, which stimulates angiogenesis, and thus to play a role in the cellular signalling involved in the tumour growth and metastatic potential of some cancers (Zhang *et al*, 1994). In ovarian cancer, Han *et al* (2006) suggested TF as an independent predictive indicator of prognosis. Conversely, Sørensen *et al* (2000) reported that survival rate was significantly lower for cancer patients with VTE than for cancer patients without VTE, and the mortality ratio associated with VTE was 2.2 for the 1-year follow-up period. The present investigation could not show the effect of TF expression or VTE development on cancer progression owing to the limited observation period. Although we can speculate that procoagulant activity and VTE affected by TF expression may cause cancer progression in ovarian cancer patients, further investigations are needed to clarify this relationship.

The influence of histological classification on TF expression was shown, particularly for clear cell carcinoma. Clear cell carcinoma is thought to display a Müllerian origin by the subsequent association with endometriosis and endometrioid carcinoma (Yoonessi *et al*, 1984). This rare tumour was recognised by the World Health Organization as a distinct histological type in 1973 and accounts for ⩽15% of all newly diagnosed ovarian cancers, and some reports suggest that clear cell carcinoma of the ovary is more likely to result in the development of VTE (Yoonessi *et al*, 1984; Pather and Quinn, 2005). Although we could not statistically confirm a prevalence effect of clear cell carcinoma, five of 11 patients with clear cell carcinoma (45%) developed pretreatment VTE, compared with 24% with non-clear cell carcinoma. The mechanism underlying this high frequency of VTE in the presence of clear cell carcinoma of the ovary are unclear. In the present investigation, clear cell carcinoma showed significantly stronger TF expression than non-clear cell carcinoma (Table 2). This suggests that the high frequency of VTE with clear cell carcinoma is likely to involve TF expression by cancer tissues.

As a limitation of this study, the small sample size might affect VTE incidence and a distribution of histological classification. And also, the small sample size might be part of the reason that we could not prove the direct relevance of clear cell carcinoma to VTE incidence. Until the relevance is proven in a next investigation, we could not recommend an intensive diagnostic test or an anti-thrombosis therapy for patients with clear cell carcinoma. To verify the relevance, further study with large number of patients with ovarian cancer will be needed. Another limitation of this study was that we could not address the alteration of TF expression after chemotherapy. We diagnosed TF expression with the surgical tissue after neoadjuvant chemotherapy for 15 patients among 32 patients. To reduce the misleading by the effect of neoadjuvant chemotherapy, we used the region containing enough amount of their original cancer cell structure for evaluation and observed more amount of TF expression from the region. Therefore, we assume cell function in the region, as well as its morphology, might not be disturbed so much by neoadjuvant chemotherapy. However, further investigation will be needed for evaluating the effect of chemotherapy on cell function.

In conclusion, this study demonstrated a relation between TF expression and clinical VTE development in patients with ovarian cancer. In particular, clear cell carcinoma has the possibility of producing excessive levels of TF and thus being more likely to develop VTE.

## Figures and Tables

**Table 1:** Patient characteristics (*n*=32)

	**Mean ± s.d.**
Age (years)	49.8 ± 13.7
BMI	23.3 ± 4.2
D-dimer (*μ*g ml^−1^)	4.92 ± 6.32
	**Number of patients**
	
*FIGO stage*	
Stage I	7
Stage II	6
Stage III	11
Stage IV	8
	
*Histological subtype*	
Serous	12
Endometrioid	4
Clear cell	11
Undifferentiated	3
Mixed epithelial	1
Yolk sac tumour	1
Underwent ultrasonography	14

BMI=body mass index; FIGO=International Federation of Gynecology and Obstetrics.

**Table 2:** Patient characteristics according to intensity of TF expression in cancer tissues

	**Intensity of TF expression**				
	**(−) *n*=5**	**(+) *n*=9**	**(++) *n*=10**	**(+++) *n*=8**	***P***
*(mean* ± *s.d.)*					
Age (years)	51.0 ± 8.1	48.8 ± 10.2	55.6 ± 14.4	42.9 ± 17.3	0.4407
BMI	25.0 ± 3.2	24.7 ± 6.1	22.7 ± 3.6	21.5 ± 1.9	0.0796
D-dimer (*μ*g ml^−1^)	1.50 ± 0.93	1.55 ± 1.06	5.28 ± 3.85	10.4 ± 9.94	0.0030
					
*Neoadjuvant chemotherapy (number of subjects)*					
(+) (*n*=15)	3	4	6	2	0.3387
					
*FIGO stage (number of subjects)*					
Stage I (*n*=7)	1	2	2	2	0.8034
Stage II (*n*=6)	1	1	2	2	
StageIII (*n*=11)	1	4	3	3	
Stage IV (*n*=8)	2	2	3	1	
					
*Histological classification (number of subjects)*					
Non-clear cell	4	8	6	3	0.0391
Clear cell	1	1	4	5	

A significant correlation existed between TF expression and D-dimer concentration (*P*=0.003; *ρ*=0.533). Tissue from clear cell carcinoma showed significantly stronger TF expression than tissue from non-clear cell carcinoma (*P*<0.05).

BMI=body mass index; FIGO=International Federation of Gynecology and Obstetrics; TF=tissue factor.

**Table 3:** Comparison of clinical characteristics (age, BMI, stage, histological classification) and intensity of TF expression between patients with VTE (*n*=10) and without VTE (*n*=22)

	**VTE (−) *n*=22**	**VTE (+) *n*=10**	***P***
*(mean*±*s.d.)*			
Age (years)	47.9 ± 13.9	54.0 ± 12.9	0.0782
BMI	24.0 ± 4.6	21.7 ± 2.4	0.9171
			
*FIGO stage (number of subjects)*			
Stage I (*n*=7)	6	1	0.1065
Stage II (*n*=6)	3	3	
Stage III (*n*=11)	8	3	
Stage IV (*n*=8)	5	3	
			
*Histological classification (number of subjects)*			
Non-clear cell (*n*=21)	16	5	0.2515
Clear cell (*n*=11)	6	5	
			
*Intensity of TF expression (number of subjects)*			
Negative (*n*=5)	5	0	0.0003
Weakly positive (*n*=9)	9	0	
Moderately positive (*n*=10)	5	5	
Strongly positive (*n*=8)	3	5	

Patients with VTE showed significantly stronger TF expression (*P*=0.0003; *ρ*=0.653).

BMI=body mass index; FIGO=International Federation of Gynecology and Obstetrics; TF=tissure factor; VTE=venous thromboembolism.
